# Selective Loss of Early Differentiated, Highly Functional PD1^high^ CD4 T Cells with HIV Progression

**DOI:** 10.1371/journal.pone.0144767

**Published:** 2015-12-17

**Authors:** Robert M. Paris, Constantinos Petrovas, Sara Ferrando-Martinez, Eirini Moysi, Kristin L. Boswell, Eva Archer, Takuya Yamamoto, David Ambrozak, Joseph P. Casazza, Richard Haubrich, Mark Connors, Julie Ake, Jerome H. Kim, Richard A. Koup

**Affiliations:** 1 Immunology Laboratory, Vaccine Research Center, NIAID, NIH, Bethesda, Maryland, United States of America; 2 Department of Microbiology and Immunology, University of Miami, Miller School of Medicine, Miami, Florida, United States of America; 3 United States Military HIV Research Program, Silver Spring, Maryland, United States of America; 4 Division of Infectious Diseases, Antiviral Research Center, University of California San Diego, San Diego, California, United States of America; 5 HIV-Specific Immunity Section, Laboratory of Immunoregulation, NIAID, NIH, Bethesda, Maryland, United States of America; 6 International Vaccine Institute, Seoul, South Korea; Jackson Laboratory, UNITED STATES

## Abstract

The role of PD-1 expression on CD4 T cells during HIV infection is not well understood. Here, we describe the differential expression of PD-1 in CD127^high^ CD4 T cells within the early/intermediate differentiated (EI) (CD27^high^CD45RA^low^) T cell population among uninfected and HIV-infected subjects, with higher expression associated with decreased viral replication (HIV-1 viral load). A significant loss of circulating PD-1^high^CTLA-4^low^ CD4 T cells was found specifically in the CD127^high^CD27^high^CD45RA^low^ compartment, while initiation of antiretroviral treatment, particularly in subjects with advanced disease, reversed these dynamics. Increased HIV-1 Gag DNA was also found in PD-1^high^ compared to PD-1^low^ ED CD4 T cells. In line with an increased susceptibility to HIV infection, PD-1 expression in this CD4 T cell subset was associated with increased activation and expression of the HIV co-receptor, CCR5. Rather than exhaustion, this population produced more IFN-g, MIP1-a, IL-4, IL-10, and IL-17a compared to PD-1^low^ EI CD4 T cells. In line with our previous findings, PD-1^high^ EI CD4 T cells were also characterized by a high expression of CCR7, CXCR5 and CCR6, a phenotype associated with increased *in vitro* B cell help. Our data show that expression of PD-1 on early-differentiated CD4 T cells may represent a population that is highly functional, more susceptible to HIV infection and selectively lost in chronic HIV infection.

## Introduction

PD-1 is expressed on the surface of T-cells, macrophages, and B cells and functions as an inhibitory co-receptor in the B7:CD28 family, specifically in the regulation of immune activation, inflammation and tolerance [1,2]. Studies of chronic viral infection have demonstrated the importance of PD-1 in the regulation of immune exhaustion in CD8 T cells, and to a lesser extent, CD4 T cells. Exhausted T cells are defined by the gradual loss of effector function, typically by decreased secretion of IFN-g, TNF-a, IL-2 cytokines, and terminal differentiation, and have been described in chronic viral infections in mice, rhesus macaques, and humans [3–6]. Interfering or blocking the PD-1 pathway can improve or restore functional CD8 T cells during chronic LCMV or SIV infection [5,7]. Recently it was also shown that blocking the PD-1/PD-L1 pathway resulted in clearance of parasitemia in a mouse model of blood-stage malaria with an increase in both CD4 T cell function and expansion of T follicular helper (T_FH_) cells and plasmablasts, indicating that this interaction is important for the development of pathogen-specific adaptive immune responses [8].

Multiple lines of evidence suggest that T cells, even those with an exhausted phenotype, may retain some functional and proliferative capacity during a chronic viral infection [9–11]. Specifically, recent evidence from adoptive transfer studies in mice show that antigen-specific CD8 T cells retain proliferative capacity, though with reduced effector function, despite an exhausted phenotype [12,13]. Another study of PD-1 expression during chronic SIV infection in Rhesus macaques demonstrated that PD-1 expression on CD4 T cells is associated with retained proliferative capacity based on *ex vivo* Ki-67 expression [14]. Taken together, these studies suggest that PD-1 expression by itself may not solely be a phenotypic marker of immune exhaustion, but may regulate subsets of T cells with a specific differentiation state and effector function, thereby limiting the inflammatory response and tissue damage during chronic infection [15].

Here, we show that in the EI CD4 T cell population there is increased expression of PD-1 relative to CTLA-4 within the subset that is CD127^high^¸ and this population is initially increased in HIV-infected compared to uninfected individuals, but then decreases concomitant with the expansion of PD-1^high^CTLA-4^high^CD127^high^ EI CD4 T cells. HIV-infected subjects with higher plasma HIV RNA had a reduced frequency of PD-1^high^ CD127^high^ EI CD4 T cells along with increased cell-associated HIV *gag* DNA in this population. Further, we demonstrate that this population with increased PD-1 expression is also associated with increased *in vitro* cytokine production, suggesting PD-1 is expressed earlier in the differentiation of CD4 compared to CD8 T cells.

## Materials and Methods

### Study subjects

HIV uninfected peripheral blood mononuclear cells (PBMC) were obtained from individuals participating in the NIH research apheresis program. Cryopreserved, HIV-infected PBMCs were obtained from three different study populations. For untreated HIV infection, cells were obtained from volunteers who participated in a therapeutic vaccination trial (no efficacy was observed) prior to receiving anti-retroviral therapy [16], who had relatively preserved CD4 counts (median 525, interquartile range [IQR] 390–879). We also obtained PBMC from HIV-infected donors with more advanced HIV (median CD4 count 148 cells/μL, IQR 59–274) participating in AIDS Clinical Trials Group study A5142 prior to initiation of combination antiretroviral therapy (cART) and at 48 weeks of therapy [17,18]. The third study population consisted of donors obtained from a cohort used to identify individuals with HIV broadly neutralizing antibodies as previously described [19]. Characteristics of these populations are provided in Table 1. All studies involving human subjects were reviewed and approved by their respective institutional review boards to include the IRB's of the National Institute of Health, National Institute of Allergy and Infectious Diseases, the University of California San Diego, and the Walter Reed Army Institute of Research. Data and stored specimens were utilized from prior multi-center clinical studies under which written informed consent was obtained for all study volunteers to store samples for future use. The use of stored samples for this study was approved the Walter Reed Army Institute of Research Institutional Review Board and the NIH/NIAID. Regarding the ACTG samples, the use of the samples and the submitted manuscript was approved from the ACTG appropriate reviewing committee. ACTG, a multi-center network, samples were collected from different sites and analyzed based on their recovery and survival. This makes impossible to identify specific IRB protocol for the used ACTG samples.

**Table 1:** Characteristics of HIV-infected subjects.

HIV-infected population		log_10_ HIV-1 RNAMean (95% CI)	CD4 countMedian (IQR)
Cohort 1 (untreated,n = 31)		3.99 (3.72–4.25)	525 (390–879)
Cohort 2 (n = 14)			
	Pre-cART	4.82 (4.45–5.18)	148 (59–274)
	48 weeks Post-cART	<1.69^1^	289 (200–743)
Cohort 3 (untreated, n = 9))		3.53 (3.23–3.78)	605 (550–821)

Abbreviations: cART, combination antiretroviral therapy; CI, confidence interval; IQR, interquartile range;

^1^ all below detection limit of 50 copies/mL

### Laboratory studies

#### Antibodies

Flow cytometry was performed using the following directly conjugated antibodies: (1) BD Biosciences: CD3-H7APC (SK7), CD45RA-Cy7PE (L48), CTLA-4-APC (BNI3), IFN-g-FITC (B27), CCR7-Alexa700 (150503), CCR5-FITC (2D7/CCR5), CCR4-PE (1G1) and IL-2-PE (MQ1-17H12), Ki67-FITC (B56); (2) Beckman Coulter: CD27-Alexa680 (IA4CD27), CD127-Cy5PE (R34.34), CD160-PE (BY55), BTLA-PE (J168-540); (3) BioLegend: PD-1-BV421 (EH12.2H7), 2B4-FITC (CD244, C1.7), IL-17a-Cy5.5PerCP (BL168), CCR6-Alexa647 (or PE, TG7/CCR6), CD27 Alexa647 (O323), CCR7 BV605 (G043H7), CXCR5 BV421 (J252D4), and CD154-Cy5PE (24–31); (4) Invitrogen: CD4-Cy5.5PE (S3.5), CD27-QD605 (CLB-27/1), CD8-QD800 (3B5). A biotinylated anti-PD-1 antibody was obtained from R&D (BAF 1086) and streptavidin-Qdot 655 was obtained from Molecular Probes. Quantum dots and Aqua amine viability dye were obtained from Invitrogen. CD27-Alexa 594, TNF-a Alexa 594, HLA-DR BV650, and CD38-Alexa 680 were conjugated in-house.

#### Polychromatic flow cytometry

For phenotypic analyses PBMCs were cultured in RPMI 1640 (Invitrogen) supplemented with 10% fetal bovine serum, 2mM L-glutamine, 100U/mL penicillin and 100 ug /mL streptomycin (Invitrogen). 1–2 x 10^6^ PBMCs were incubated with Aqua viability dye and surface stained with titrated amounts of antibodies to panel (1): CD3, CD4, CD8, CD27, CD45RA, CD127, PD-1, 2B4, CD160 followed by intracellular staining for CTLA-4; (2) CD3, CD4, CD8, CD27, CD45RA, CD127, PD-1, CCR4, CCR5, CCR6, and CCR7; or (3) CD3, CD4, CD8, CD27, CD45RA, CD127, PD-1, BTLA, HLA-DR, and CD38; (4) CD3, CD4, CD27, CD45RA, CD127, CCR7, PD-1, CXCR5, CCR6 followed by intracellular staining for CTLA-4. Cells were then washed and fixed with 1% paraformaldehyde prior to event collection. In some experiments Ki67 was also used during the ICS staining. Given the relatively small size of the described parental populations, i.e. the PD-1^high^ CTLA-4^high^ CD4 T cells, analysis of the proliferation profile is presented only for samples whith reasonable anticipated populations. For intracellular cytokine staining (ICS) 3 x 10^6^ PBMCs were rested for 2h and incubated in 1mL of medium containing brefeldin A (10ug/mL) in the absence or presence of HIV-1 Gag-peptide pools (15mers overlapping by 11 residues; National Institutes of Health AIDS Research and Reference Reagent Program), or 1 ug/mL SEB (Sigma) for 6 hours. After washing, cells were surface stained with Aqua, CD4, CD8, CD27, CD45RA, CD127, and PD-1, washed and incubated with fluorescent-conjugated streptavidin (for biotinylated PD-1). Cells were then washed again, permeabilized (Cytofix/Cytoperm kit; BD Biosciences), and stained with antibodies to CD3, IFN-g, IL-2, IL-17a or TNF-a, IL-2 and CTLA-4. After fixation with 1% paraformaldehyde, events were collected on a modified LSRII flow cytometer (BD Immunocytometry Systems). Electronic compensation was performed with antibody capture beads (BD Biosciences) stained separately with antibodies used in the test samples. Data were analyzed using FlowJo Version 9.6 (TreeStar, Ashland, OR).

#### Measurement of *in vitro* cytokine production

Fresh PBMCs (2 x 10^8^) obtained from HIV-uninfected leukapheresis subjects were sorted after Ficoll separation. After incubation with Aqua viability dye, cells were stained with antibodies to CD4, CD8, CD19, CD14, CD27, CD45RA, PD-1, BTLA and CD127. CTLA-4 was not included due to the requirement for cell permeabilization. Four populations (CD27^high^CD45RA^high^, CD27^low^CD45RA^high/low^, PD-1^high^CD127^high^CD27^high^CD45RA^low^ and PD-1^low^CD127^high^CD27^high^CD45RA^low^) of 0.5x10^6^ PBMCs were sorted and stimulated with plate-bound anti-CD3 stimulation (10 mcg/mL, BD Pharmingen, clone UCHT1) and co-stimulatory anti-CD28/49d (1.3 mcg/mL, BD Fastimmune). Supernatants were harvested after overnight stimulation for subsequent cytokine (IFN-g, TNF-a, IL-2, IL-4, IL-5, IL-10, and IL-17) quantification using Luminex technology according to the manufacturer’s instructions (Milliplex MAP Kit, Cat. No. HCYTOMAG-60K, Millipore).

#### Telomerase activity

From the same sorting experiment and populations used for in vitro cytokine production, 30,000 cells each were sorted and lysed using the Quantitative Telomerase Detection (QTD) lysis buffer for telomerase activity, which was performed using the QTD Kit according to manufacturer’s instructions (Allied Biotech, Vallejo, CA).

#### HIV-1 Gag DNA PCR

Similarly, approximately 5000 cells were sorted from cryopreserved, HIV-infected PBMC’s directly into lysis buffer and quantification of HIV *gag* DNA was performed by quantitative PCR (qPCR) by means of a 5′ nuclease (TaqMan) assay with an ABI 7700 system (Perkin Elmer, Norwalk, CT) as previously described [20,21]. Standards were constructed for absolute quantification of *gag* and albumin copy number and were validated with sequential dilution of 8E5 cell lysates that contain one copy of *gag* per cell. Duplicate reactions were run and template copies calculated using ABI7700 software.

#### *In vitro* HIV infection

Sorted memory CD4 T cells from two healthy donors were subjected to *in vitro* infection with a R5-tropic EGFP HIV-1 AD8 at multiplicity of infection (MOI) of 0.01 for 5 days. Cells were harvested on day 5 and PD-1 levels in non-infected (GFP-) and cells harboring virus (EGFP+) were analyzed by flow cytometry.

### Statistical analysis

Experimental variables were analyzed using nonparametric statistical tests of inference: Mann-Whitney U test, the Wilcoxon matched-pairs signed rank test, or the Kruskal-Wallis test with Dunn’s multiple comparison post-test as appropriate. Correlation analysis was performed using the nonparametric Spearman test. The Generalized Estimating Equations regression analysis was utilized to model longitudinal measurements of HIV-1 viral RNA or CD4 count to account for the non-independence of intra-individual repeated measures. Statistical analyses were performed with GraphPad Prism (GraphPad Software, version 5.0) or Stata Statistical Software, Release 11 (StataCorp, College Station, TX).

## Results

### Loss of PD-1^high^CTLA-4^low^ early-differentiated CD4 T cells in advanced HIV infection

**Fig 1:**
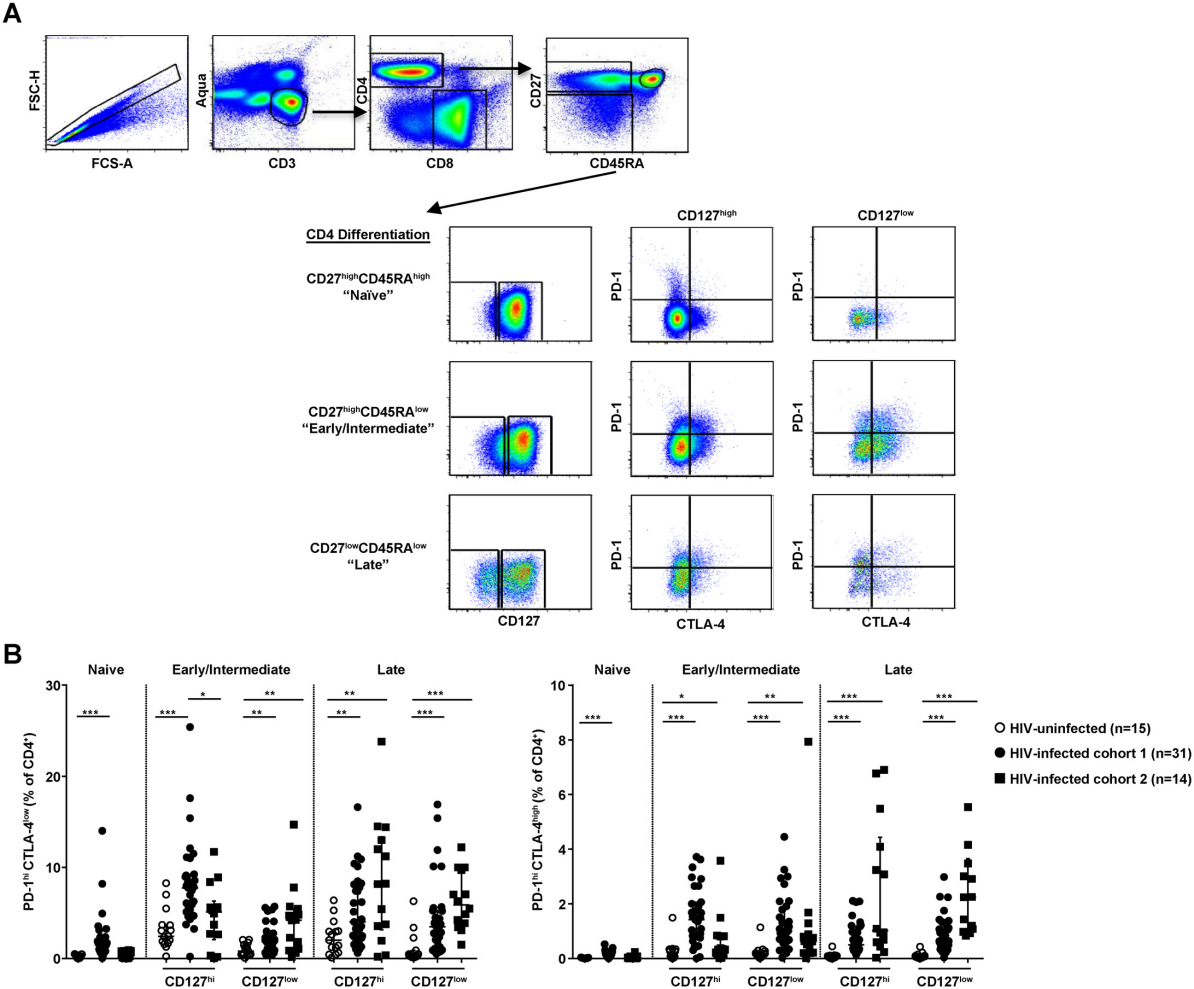
The frequency of less differentiated PD-1^high^ CD127^high^ CD4 T cells is reduced compared with more differentiated subsets in advanced HIV infection. **(A)** Gating strategy to define differentiation status of CD127, PD-1 and CTLA-4 expression by CD4 T cells. Differentiation was defined by gating on CD27 and CD45RA with CD27^high^CD45RA^high^ (referred to as **Naïve**), CD27^high^CD45RA^low^ (**Early/Intermediate**), and CD27^low^CD45RA^low^ (**Late**). **(B)** Distribution plots from HIV- infected subjects compared to HIV-uninfected (open circles, n = 15) from two cohorts with HIV infection: Cohort 1 (median CD4 count 525 cells/μl, filled circles, n = 31); and Cohort 2 with more advanced infection (median CD4 count 148 cells/μl, filled squares, n = 14) of PD-1 and PD-1/CTLA-4 expression by differentiation status and CD127 (IL-7Ra) staining demonstrating an altered/reduced frequency of PD-1^high^ CTLA-4^high/low^ CD127^high^ CD4 T cells of early/intermediate differentiation compared to more differentiated subsets which show increased PD-1 expression with more advanced HIV infection. Plots include median and interquartile range, *p< 0.05, **p< 0.001, ***p< 0.0001 by Kruskal-Wallis or Mann-Whitney test.

First, we determined the expression patterns for two major co-inhibitory receptors for CD4 T cells from HIV uninfected (n = 15) and untreated HIV-infected subjects from a cohort with earlier HIV infection (Cohort 1, n = 31, median CD4 count 525 cells/μl) or with more advanced disease prior to treatment (Cohort 2, n = 14, median CD4 count 148 cells/ μl) (Fig 1A and Table 1). Skewing of maturation subsets was evident by a significant lower frequency of CD27^high^CD45RA^high^ (naïve) and CD27^high^CD45RA^low^ CD4 T cells associated with an increased frequency of CD27^low^CD45RA^low^ (late differentiated, LD) CD4 T cells in Cohort 2 with lower median CD4 count, consistent with more advanced disease (S1A Fig). Although several markers have been used to define memory phenotypes, we found distinct patterns of PD-1 and CTLA-4 (two major regulators of T cell activation and function) expression in T cell populations dependent on CD127, the IL-7 receptor (Fig 1A and 1B). Overall, the relative frequency of cells expressing a PD-1^high^CTLA-4^low^ phenotype was increased in HIV-infected compared to uninfected individuals, in all CD4 T cell memory subsets tested (Fig 1B). A strong association was found between the expression of PD-1 and differentiation of CD4 T cells in donors *with less advanced disease* (Cohort 1): the CD127^high^ EI compartment had the highest frequency of PD-1^high^ CD4 T cells (Fig 1B) (p < 0.0001, for PD-1^high^CTLA-4^low^ in CD127^high^ vs. CD127^low^ EI and p = 0.268 for PD-1^high^CTLA-4^low^ CD127^high^ vs. CD127^low^ in the LD compartments respectively). A significantly lower frequency of PD-1^high^CTLA-4^low^CD127^high^ EI CD4 T cells was found in the cohort with more advanced disease (Cohort 2), a pattern that was not seen in the other memory populations. On the other hand, the frequency of PD-1^high^CTLA-4^high^ cells was decreased, although not significantly, in both CD127^high^ and CD127^low^ EI CD4 T cells from HIV-infected individuals with more advanced disease (Fig 1B).

The expression pattern of these receptors appeared to differ from CD8 T cells from HIV-infected and uninfected donors (S1C Fig), with differences in PD-1^high^CTLA-4^low^ frequencies noted for more differentiated CD8 T cells (CD127^low^). Interestingly, the comparison between PD-1^high^ and PD-1^high^ CTLA-4^high^ expression profiles on CD27^high^CD45RA^low^ CD4 T cells from HIV negative individuals indicates that PD-1 is up-regulated prior to CTLA-4 during CD4 T cell differentiation. Minimal expression of other negative co-stimulatory molecules (2B4, CD160) on CD4 T as compared to CD8 T cells was found (data not shown). These observations indicate that the regulation of both PD-1 and CTLA-4 differs between CD4 and CD8 T cells, particularly in early-differentiated CD4 (CD8) T cell populations.

**Fig 2:**
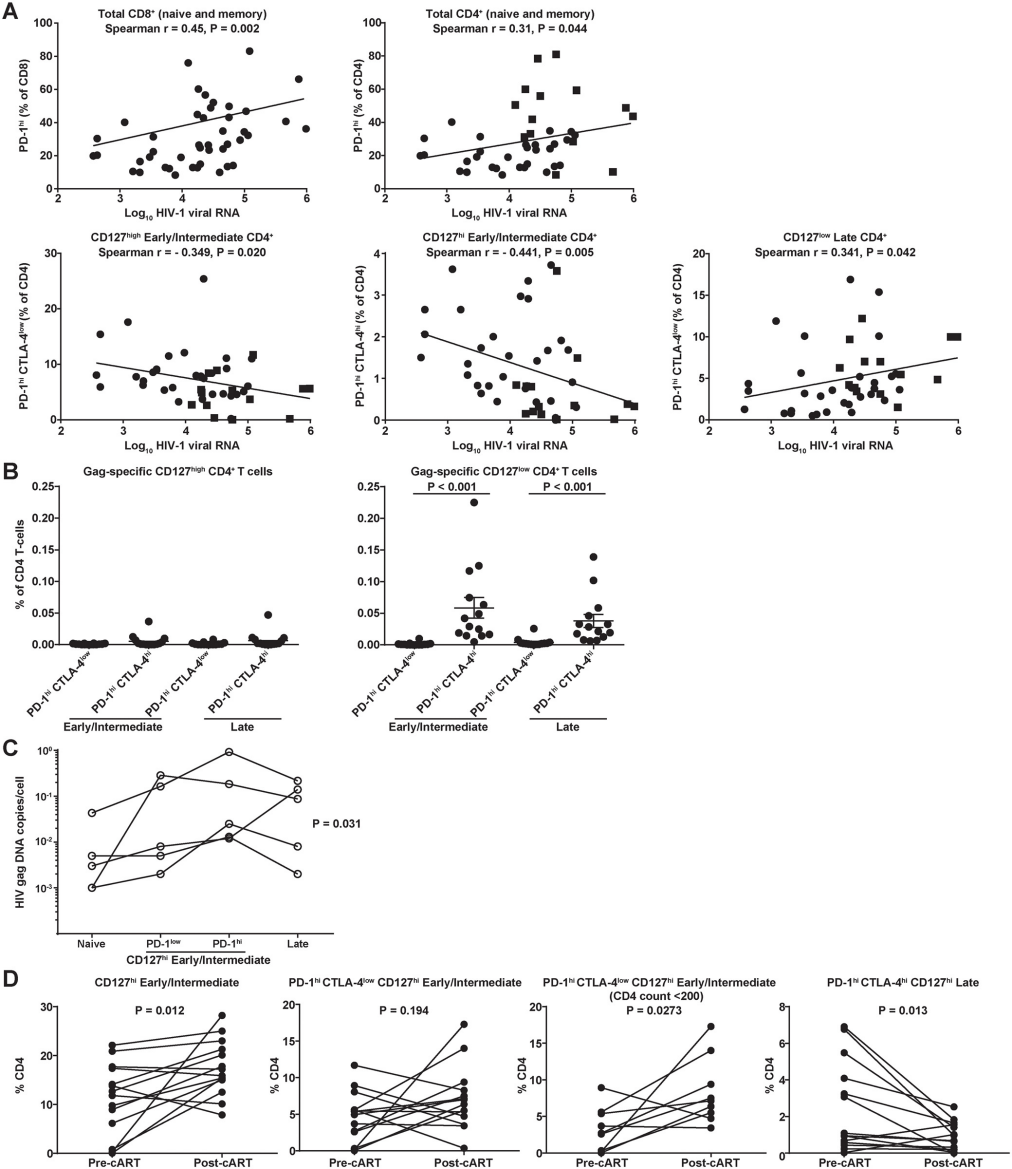
Seletctive loss of PD-1^high^CTLA-4^low/high^CD127^high^ Early/Intermediate CD4 T cells occurs with higher plasma HIV-1 viral RNA levels and higher cell-associated viral DNA. **(A)** Scatter plots of HIV-1 viral RNA and fitted regression lines for total (naïve and memory) CD8 and CD4 T cells demonstrating increased PD-1 expression with higher viral replication. However, for CD4 T cells of Early/Intermediate differentiation expressing CD127 and PD-1 or PD-1/CTLA-4 there is a negative association compared with more differentated (CD127^low^) CD4 T cells. Spearman rank correlation coefficients and associated p-values are shown. **(B)** Donors (n = 14, five from Cohort 1 and nine from Cohort 3) with HIV Gag-specific CD4 T-cell responses are more differentiated (CD127^low^) and co-express both PD-1 and CTLA-4. **(C)** Cell-associated HIV-1 *gag* DNA (no. copies/cell) for sorted T cell populations (see S3 Fig for gating strategy). Individual differences between differentiation subsets (shown for each individual by a connecting line) are statistically significant (p = 0.031 by Friedman test). **(D)** PD-1^high^CTLA-4^low^CD127^high^ Early/Intermediate CD4 T cells are increased after antiretroviral therapy. Relative frequencies of bulk CD4 populations before and after initiation of combination antiretroviral therapy (cART). Connected symbols represent pre-cART and 48 weeks post-cART (Cohort 2, n = 14, Wilcoxon matched-pairs test, p-values shown in figure). The PD-1^high^CTLA-4^low^ CD127^high^ group is analyzed separately for subjects who started cART with a CD4 count less than 200.

To further investigate the impact of HIV infection on the described CD4 T cell populations, we combined data for HIV-infected subjects from Cohorts 1 and 2, before treatment (n = 45). Consistent with previous studies, we found increased frequencies of PD-1^high^ cells in total (naïve and memory) CD4 and CD8 T cell compartments with higher viral load (Fig 2A). However, this association was stronger in the case of CD8 T cells (Spearman r = 0.45, p = 0.002) compared with CD4 T cells (r = 0.31, p = 0.044) (Fig 2A), where higher viral load was correlated with higher PD-1 expression on more differentiated, Late (CD127^low^ CD27^low^CD45RA^low^) CD4 T cells (Spearman r = 0.341, p = 0.042) (Fig 2A). However, PD1^high^CD127^high^ EI subset was negatively associated with viral load, independent of the expression of CTLA4 (Fig 2A).

To extend these findings, we assessed the association of the EI CD4 T cell phenotype with longitudinal viral load measurements in a GEE regression analysis in which repeated measurements HIV-1 viral RNA were modeled as the dependent variable. We observed a slight decline in HIV-1 viral load over time associated with the PD-1^high^ CTLA-4^low^ CD127^high^ EI CD4 T cell phenotype (regression coefficient = -0.062, p = 0.023), which was the only statistically significant phenotypic association with longitudinal viral load (other data not shown). We further examined untreated subjects for HIV Gag-specific responses (S2 Fig) as HIV-specific CD4 T cells have been shown to be preferentially infected [21]. HIV-specific CD4 T cells expressed an IFNg^+^IL-2^-^ profile (S2 Fig). Among 14 subjects with a detectable HIV Gag-specific CD4 response, we found that the majority of Gag-specific CD4 T cells (median 65.0%, range 18.4 to 94.4%) were more differentiated (CD127^low^) and co-expressed PD-1 and CTLA-4 (Fig 2B), which is consistent with prior studies [22,23].

No down-regulation of PD-1 was observed with *in vitro* HIV infection of sorted, memory CD4 T cells in infected compared to uninfected cells (S3A Fig). Our data indicate that PD-1^high^CD127^high^ EI CD4 T cells may be preferentially lost during chronic HIV infection. Furthermore, the very low frequency of HIV-specific CD4 T cells challenges their importance for CD4 dynamics within this early-differentiated compartment.

### Restoration of PD-1^high^CTLA-4^low^ early-differentiated CD4 T cells after antiretroviral therapy

Our data imply that the progressive loss of PD-1^high^ CD4 T cells in an early differentiation state could be mediated, at least in part, by increased infection by HIV. To confirm this, we evaluated sorted CD4 T cell populations (Fig 2C) and observed an increase in the frequency of HIV-1 *gag* DNA (n = 5 donors) in the PD-1^high^ compared to PD-1^low^ populations. Since cell-associated DNA content will vary with plasma viral RNA, we compared paired differences in HIV-1 *gag* DNA across differentiation subsets which were statistically significant (p = 0.031, Friedman test), consistent with the interpretation that early differentiation is associated with increased HIV infection. Only the difference between the PD-1^high^ CD127^high^ EI and naïve compartments was statistically significant after Dunn’s multiple comparisons correction (p < 0.05). Interestingly, a comparable frequency of HIV-1 *gag* DNA copies was observed between the PD-1^high^CD127^high^EI and LD CD4 T cell compartments (Fig 2C).

In the HIV treatment cohort (Cohort 2, Table 1) in which PBMC were obtained before and 48 weeks after initiation of combination antiretroviral therapy (cART), we observed an increase of the relative frequency of CD4 T cells expressing high levels of CD127 with therapy as previously described [24] (Fig 2D). Consistent with their increased loss during advanced disease (Fig 1C), a significant expansion (p = 0.0273, Wilcoxon matched-pairs test) of the PD-1^high^CTLA-4^low^CD127^high^ EI CD4 T cell subset was found in subjects with lower CD4 counts (<200 cells/μL) at treatment initiation (Fig 2D). In contrast, cART led to a decreased frequency of PD-1^high^CTLA-4^high^ cells, especially in theCD127^high^ LD CD4 T cell compartment (p = 0.013), as well as within the respective CD127^low^ populations (data not shown). Although the data shown are relative frequencies and not absolute counts, there is the possibility of redistribution of memory subsets following cART initiation. Taken together, our data indicate that the dynamics of early-differentiated CD4 T cells could be regulated by infection-depletion along with other mechanisms that could promote their differentiation towards more mature CD4 T cell phenotypes.

### PD-1 up-regulation in early-differentiated CD4 T cells is associated with increased activation and expression of the HIV co-receptor CCR5

**Fig 3:**
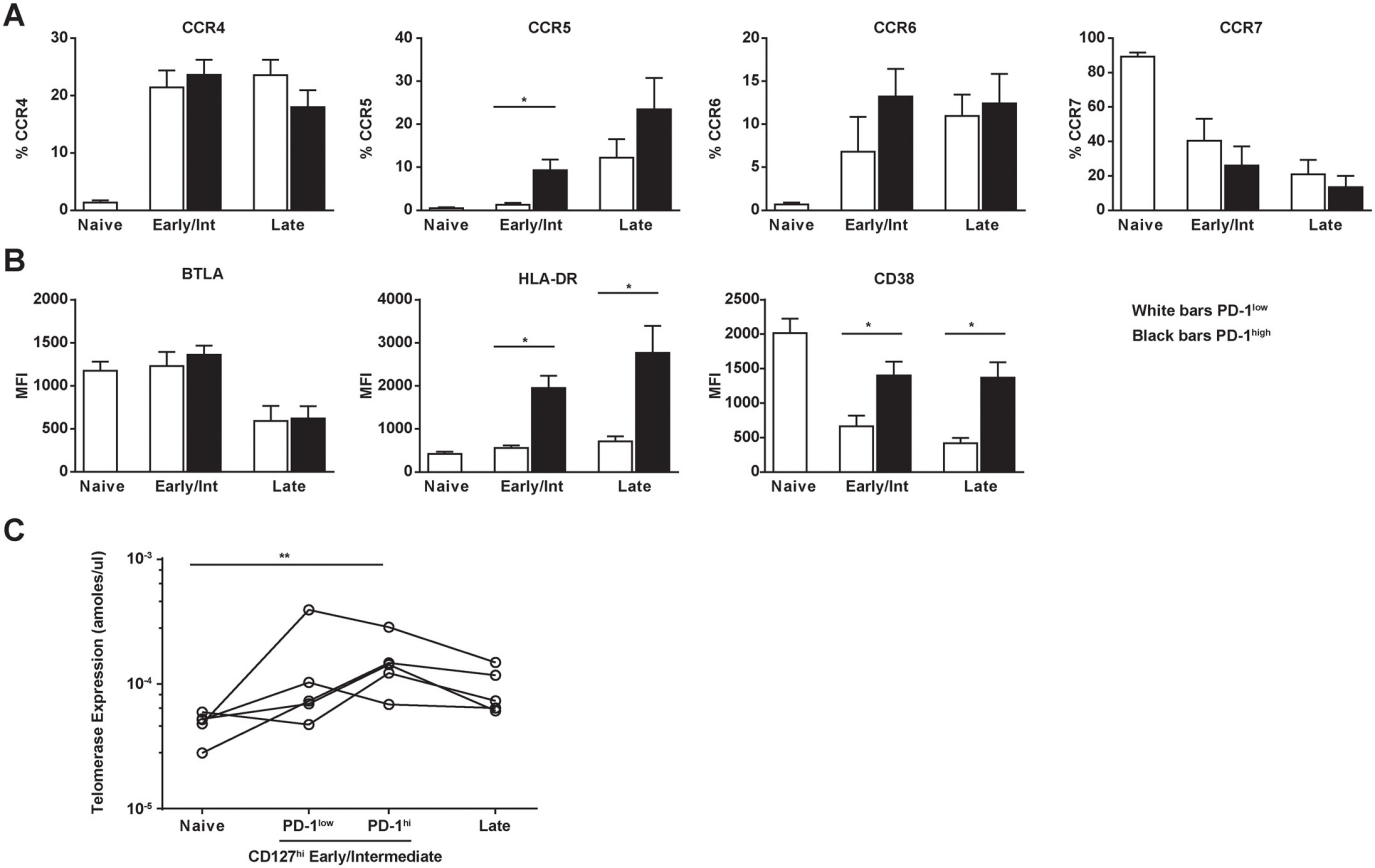
PD-1^high^CD127^high^ Early/Intermediate CD4 T cells express the HIV coreceptor CCR5, activation markers HLA-DR and CD38, and demonstrate evidence of TCR stimulation. **(A)** Bar graphs showing the relative frequency of CD4 T cell populations expressing several chemokine receptors (CCR4, CCR5, CCR6, and CCR7) and **(B)** markers of activation/differentiation (BTLA, HLA-DR, and CD38) (n = 7 HIV-infected donors). All populations are CD127^high^. MFI, mean fluorescence intensity; bars represent mean and SEM, *p< 0.05, after correction by Dunn’s multiple comparisons test. (**C**) Evidence of recent TCR stimulation was assessed based on telomerase expression by qRT-PCR assay of sorted populations (see S3 Fig for gating strategy). Individual differences between differentiation subsets (shown for each individual by a connecting line) were statistically significant (p = 0.02, Friedman test).

Next, we investigated whether expression of PD-1 in CD127^high^ EI T cells was associated with differential expression of chemokine receptors, particularly CCR5 or activation markers, parameters directly associated to HIV infectivity. We performed further CD4 T cell phenotypic analysis for the chemokine receptors CCR4, CCR5, CCR6, and CCR7 as well as the activation markers CD38, HLA-DR, and BTLA, a co-inhibitory receptor with functional characteristics similar to PD-1 and CTLA-4, which is increased among less differentiated T cells [25]. PD-1^high^ CD127^high^ EI CD4 T cells had significantly higher CCR5 expression Compared to PD-1^low^ CD127^high^ EI CD4 T cells (Fig 3A), but did not show any other significant differences in chemokine receptor expression (Fig 3A). Increased expression of activation markers per cell (judged by Mean Fluorescence Intensity-MFI) was observed with increased differentiation. However, both HLA-DR and CD38 expression was significantly up-regulated in the PD-1^high^ cells in the early differentiated CD27^high^CD45RA^low^ compartment and CD27^low^CD45RA^low^CD4 T cells (Fig 3B). We observed similar patterns of chemokine and activation marker expression in the CD127^low^ compartment with increasing differentiation (data not shown) where there were significant differences in expression of CCR5, HLA-DR, and BTLA between PD-1 high and low in the EI CD4 T cell population. To assess whether this represents actual T cell activation rather than up-regulation of PD-1 by other bystander mechanisms [26], we characterized several sorted populations for telomerase activity. We found an overall increased telomerase activity with differentiation (p = 0.02), but the PD-1^high^CD127^high^ EI population was the only differentiation phenotype significantly different from the naïve population (p < 0.05 after Dunn’s multiple comparisons correction, Fig 3C). This suggests that PD-1^high^CD127^high^ EI CD4 T cells represent an early state of CD4 T cell differentiation that have received activation signals by TCR engagement [27,28]. Our data suggest that increased expression of CCR5 on an activated background could result in the increased susceptibility of PD-1^high^CD127^high^ EI CD4 T cells to HIV infection.

### PD-1^high^ early-differentiated CD4 T cells are characterized by increased functionality

**Fig 4:**
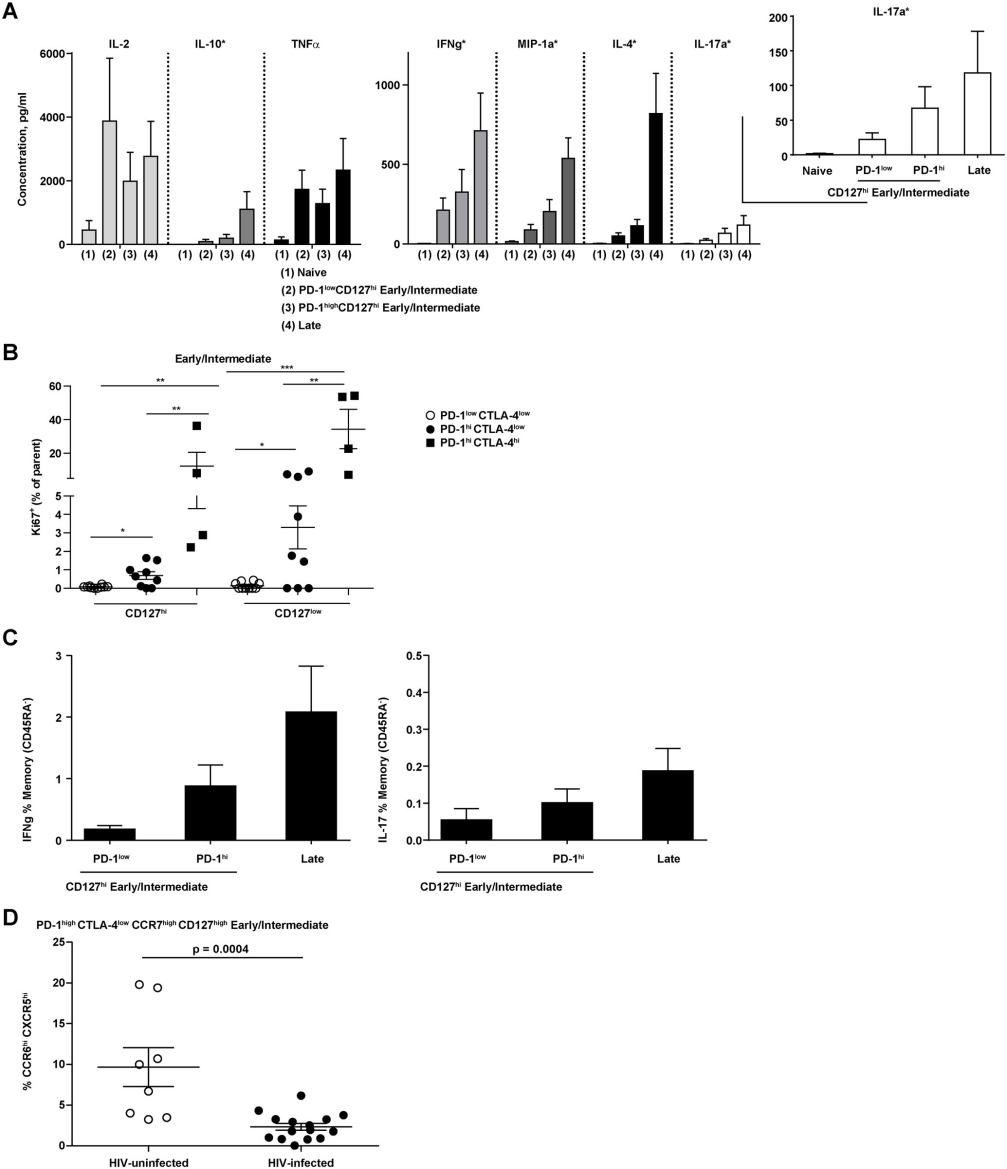
PD-1^high^CD127^high^ Early/Intermediate CD4T cells maintain broad cytokine production. **(A)** Cytokine production after polyclonal stimulation (anti-CD3 with anti-CD28 and anti-CD49d co-stimulation) measured by bead-based Luminex technology of fresh, sorted CD4 T cells from HIV-uninfected donors (n = 5, *p< 0.05 by Friedman test for each cytokine across cell populations). **(B)** Percent Ki67^+^ staining cells for CD127^high^ and CD127^low^ early/intermediate CD4 T cells from HIV-infected Cohort 1 (n = 11). **(C)** Differentiation phenotype of IFN-g or IL-17a positive cells detected after (6h) *ex vivo* SEB stimulation for HIV-infected donors (n = 5). No differences were statistically significant (Mann-Whitney test) **(D)** The relative frequency of the CCR6^high^CXCR5^high^ population within the CCR7^high^PD-1^high^CD127^high^ Intermediate CD4 T cell population is decreased in HIV-infected (n = 15) compared to uninfected (n = 8) individuals (p = 0.0004, Mann-Whitney test).

Given the role of PD-1 in CD8 T cell exhaustion during chronic viral infections [29] we sought to investigate whether PD-1^high^CD127^high^ EI CD4 T cells were characterized by impaired functionality, a hallmark of the exhaustion phenotype. To this end, we used sorted CD4 (S3B Fig) T cell populations from uninfected individuals and examined the effect of *in vitro* TCR stimulation using a functional plate-bound anti-CD3 antibody and measuring cytokine production in the supernatants. We observed increased cytokine production (IFN-g, MIP1-a, IL-4, IL-10, and IL-17) from naïve to PD-1^low^, PD-1^high^CD127^high^ EI and LD CD4 T cells (p = 0.0026, Kruskal-Wallis test, Fig 4A). Interestingly, a considerable production of IL-17 between PD-1^high^CD127^high^ EI and LD CD4 T cells was found (Fig 4A) underlining the possible impact of the loss of this particular early-differentiated CD4 T cell population in the overall compromised of IL-17-mediated defense mechanisms in chronic HIV [30]. In addition, there was evidence of proliferative capacity with increased Ki-67 expression in the CD127high and CD127low EI populations with expression of PD-1 and CTLA-4 (Fig 4B, S3C Fig). We then used staphylococcal enterotoxin B (SEB) for polyclonal stimulation to assess, using a flow cytometry assay, *ex vivo* production of IFN-g and IL-17 in untreated HIV-infected individuals (n = 5) (Fig 4B, S3C Fig). We observed a similar pattern of cytokine secretion compared with anti-CD3 stimulation (Fig 4B). These results indicate the possible impact of the loss of EI CD4 T cell population in the overall compromised defense mechanisms of chronic HIV patients [30].

We have previously shown that CD4 T cells expressing a CCR7^high^CXCR5^high^CCR6^high^PD-1^high^ phenotype can provide increased *in vitro* B cell help and is decreased in HIV infection [31]. Here, we further examined this “peripheral T_FH_^”^ phenotype and found that within the PD-1^high^CD127^high^CCR7^high^ EI CD4 T compartment, a subset of CD4 T cells with increased HIV gag DNA content (Fig 2C), there is a significant loss of CXCR5^high^CCR6^high^ cells (Fig 4C, S3D Fig). Taken together, these data indicate that PD-1 expression in early-differentiated CD4 T cells may not be associated with functional defects and exhaustion of this less differentiated population.

## Discussion

PD-1 is considered to play an important role in the regulation of the CD-4 T cell response due to its increased expression in virus-specific CD4 T cells [22]. A large body of evidence has established that PD-1 is a critical mediator of CD8 T cell exhaustion. Up-regulation of PD-1 in antigen-specific CD8 T cells results in decreased proliferative and effector capacities in chronic viral infection [4,5,32] and cancer [33]. However, the role of PD-1 as a regulator of CD4 T cell function is not well understood. Here, we demonstrate that the pattern of PD-1 expression differs between bulk CD4 and CD8 T cells for both HIV-infected and uninfected individuals indicating a differential regulation of the receptor on CD8 T cells compared to CD4 T cells either at the transcription level or due to the sensitivity of extracellular/intracellular signals regulating the surface expression of PD-1. Analysis of the co-expression of PD-1 and CTLA-4 in early-differentiated (CD127^high^CD27^high^CD45RA^low^) CD4 T cells from uninfected subjects revealed that PD-1 is expressed earlier than CTLA-4 during the differentiation of CD4 T cells (p < 0.0001). In agreement with previous studies [22,23], the majority of HIV Gag-specific cells express a more differentiated phenotype (CD127^low^) skewed towards PD-1^high^CTLA-4^high^, especially in the late-differentiated “effector memory” compartment (CD27^low^CD45RA^low^) where a minority of bulk CD4 T cells is characterized by a PD-1^high^CTLA-4^low^ phenotype. However, the inclusion of CTLA-4 in our analysis revealed an altered dynamic between PD-1^high^CTLA-4^low^ and PD-1^high^CTLA-4^high^ early during the differentiation of CD4 T cells and with respect to HIV progression.

We further analyzed the phenotype and function of PD-1^high^CTLA-4^low^CD127^high^ EI CD4 T cells and found that PD-1 expression was associated with increased activation, measured by HLA-DR and CD38 expression and increased susceptibility to HIV infection, based on the expression of the HIV co-receptor CCR5. These cells are also characterized by increased telomerase activity (Fig 3C), suggesting that this population represents an early state of CD4 T cell differentiation that have been preferentially activated by TCR engagement [27,28]. Furthermore, PD-1^high^ expression marks higher sensitivity to *in vitro* spontaneous and CD95/Fas-induced apoptosis in less differentiated CD4 T cells, although in significantly lower levels compared to PD-1^high^ “effector” CD4 T cells (data not shown). Accordingly, a decreased frequency of PD-1^high^CTLA-4^low^CD127^high^ EI CD4 T cells was associated with increased HIV-1 viral load over time. This observation and the demonstration of increased HIV *gag* DNA in this compartment suggest increased susceptibility to HIV infection which is in line with previous studies, where HIV infection was analyzed in CD127^high^ CD4 T cells [23]. We should emphasize that *in vitro* infection of CD4 T cells was not associated with any down-regulation of PD-1 in infected compared to uninfected cells. Hence, we hypothesize that increased infection and depletion could affect the dynamics of early-differentiated CD4 T cells along with other mechanisms that promote their differentiation towards mature CD4 T cell phenotypes. More importantly, the contribution of PD-1^high^CTLA-4^low^CD127^high^ EI CD4 T cells to the establishment of a latent HIV-1 reservoir compared to highly differentiated CD4 T cell populations (for example, effector PD-1^high^ cells) which are more susceptible to cell death should be investigated further. Collectively, our data suggest that these early-differentiated CD4 T cells may be more susceptible to HIV infection due to increased activation and increased co-expression of CCR5 and PD-1. We propose that the dynamics of CD4 T cells may be altered by their susceptibility to HIV infection (PD-1^high^CD127^high^CD4 T cells) and the skewed maturation of HIV-specific CD4 T cells (PD-1^high^CD127^low^), which are preferentially infected and highly sensitive to viral load changes and TCR stimulation.

Interestingly, we found that PD-1^high^ early-differentiated T cells were capable of producing a wide range of cytokines with overall cytokine production higher in PD-1^high^ compared to PD-1^low^ cells from the CD127^high^ EI CD4 T cell compartment among HIV-uninfected donors. This is consistent with the observation in the Rhesus macaque SIV model in which PD-1 expression on CD4 T cells, although not defined by differentiation phenotype, had retained proliferative capacity [14]. Hence, PD-1 signaling in the CD4 T cell compartment does not necessarily appear to confer an “exhaustion” status [14]. A loss of CD4 T cells producing IL-17 in HIV infected individuals has been previously described [34,35]. Our data indicate that the decline of PD-1^high^CTLA-4^low^CD127^high^ EI CD4 T cells, mediated, at least in part, by increased susceptibility to HIV infection, could contribute to the loss of IL-17+ CD4 T cells even at a very early step of CD4 T cell differentiation. Similarly, we observed that this phenotype overlaps with a circulating “T_FH_” phenotype, which was decreased in HIV-infected subjects, consistent with our previous study [31]. Previous studies have shown that CD4 T_FH_ cells within lymph nodes may be the major reservoir for HIV infection and replication [36]. Whether increased HIV *gag* DNA in circulating PD-1^high^CTLA-4^low^CD127^high^ EI CD4 T cells reflects increased infection of a particular follicular CD4 T cell population within the lymph node needs further investigation. Together, our data show an accelerated expression of PD-1 in the early differentiation of CD4 T cells that is associated with increased cytokine production as opposed to an expected decrease in cytokine response observed with PD-1 expression [4].

Overall, our data indicate that PD-1 and CTLA-4 could serve as a very early marker of differentiation of CD4 T cells during HIV infection marking cells with increased sensitivity to infection. In contrast to CD8 T cells, our data suggest that a functional restoration of CD4 T cells in HIV possibly requires the manipulation of PD-1 as well as other co-inhibitory receptors, like CTLA-4.

## Supporting Information

S1 Fig **(A)** Distribution plots showing skewed CD4 differentiation of HIV- infected subjects compared to HIV-uninfected (open circles, n = 15) from two cohorts with HIV infection: Cohort 1 (median CD4 count 525 cells/μl, filled circles, n = 31); and Cohort 2 with more advanced infection (median CD4 count 148 cells/μl, filled squares, n = 14). **(B)** Representative flow cytometry plots and the gating strategy used to characterize CD4 and CD8 T cell populations. **(C)** Comparative plots of PD-1 and PD-1/CTLA-4 expression by differentiation status and CD127 staining for populations of **Naive** (CD27^high^ CD45RA^high^), **Early/Intermediate** (CD27^high^CD45RA^low^) and **Late** (CD27^low^ CD45RA^low^) CD8 T cells from HIV-uninfected (open circles, n = 9) and HIV-infected (filled circles, n = 31) subjects. *p< 0.05, **p< 0.001, ***p< 0.0001 by Mann-Whitney test.
(TIFF)

S2 Fig Representative flow cytometry, gating strategy and overlay plots of Gag-specific, IFN-g-producing CD4 and CD8 T-cells for specific populations is shown.
(TIFF)

S3 Fig **(A)** Sorted memory (Early/Intermediate, CD27^high^ CD45RA^high^) CD4 T cells from two healthy donors were subjected in vitro HIV infection. PD-1 levels in non-infected (EGFP-) and cells harboring virus (EGFP+) were analyzed by flow cytometry. **(B)** Gating strategy for sorting PD-1^high^CD127^high^ Early/Intermediate and other CD4 T cell populations. Due to the requirement for surface staining, intracellular anti-CTLA-4 was not included as sorting parameter. **(C)** Percent Ki67^+^ staining cells for CD127^high^ and CD127^low^ naïve and late CD4 T cells from HIV-infected Cohort 1 (n = 11). Not all populations for all donors are plotted due to the small population size.. **(D)** Representative flow cytometry, gating strategy and overlay plots after polyclonal stimulation with SEB for IFN-g or IL-17 (shown) producing CD4 T-cells for specific populations is shown. **(E)** Representative flow cytometry plot and gating strategy demonstrating loss of CD127^high^CCR7^high^PD-1^high^CTLA-4^low^ CXCR5^high^CCR6^high^ Early/Intermediate CD4 T cells with HIV infection.
(TIFF)

## References

[1] Sharpe AH, Wherry EJ, Ahmed R, Freeman GJ (2007) The function of programmed cell death 1 and its ligands in regulating autoimmunity and infection. Nat Immunol 8: 239–245. 17304234 10.1038/ni1443

[2] Kulpa DA, Lawani M, Cooper A, Peretz Y, Ahlers J, et al (2013) PD-1 coinhibitory signals: the link between pathogenesis and protection. Semin Immunol 25: 219–227. 10.1016/j.smim.2013.02.002 23548749 PMC3795833

[3] Petrovas C, Casazza JP, Brenchley JM, Price DA, Gostick E, et al (2006) PD-1 is a regulator of virus-specific CD8+ T cell survival in HIV infection. J Exp Med 203: 2281–2292. 16954372 10.1084/jem.20061496 PMC2118095

[4] Day CL, Kaufmann DE, Kiepiela P, Brown JA, Moodley ES, et al (2006) PD-1 expression on HIV-specific T cells is associated with T-cell exhaustion and disease progression. Nature 443: 350–354. 16921384 10.1038/nature05115

[5] Barber DL, Wherry EJ, Masopust D, Zhu B, Allison JP, et al (2006) Restoring function in exhausted CD8 T cells during chronic viral infection. Nature 439: 682–687. 16382236 10.1038/nature04444

[6] Wherry EJ, Ha SJ, Kaech SM, Haining WN, Sarkar S, et al (2007) Molecular signature of CD8+ T cell exhaustion during chronic viral infection. Immunity 27: 670–684. 17950003 10.1016/j.immuni.2007.09.006

[7] Velu V, Titanji K, Zhu B, Husain S, Pladevega A, et al (2009) Enhancing SIV-specific immunity in vivo by PD-1 blockade. Nature 458: 206–210. 10.1038/nature07662 19078956 PMC2753387

[8] Butler NS, Moebius J, Pewe LL, Traore B, Doumbo OK, et al (2012) Therapeutic blockade of PD-L1 and LAG-3 rapidly clears established blood-stage Plasmodium infection. Nat Immunol 13: 188–195. 10.1038/ni.2180 PMC3262959 22157630

[9] Roederer M, Dubs JG, Anderson MT, Raju PA, Herzenberg LA, et al (1995) CD8 naive T cell counts decrease progressively in HIV-infected adults. The Journal of Clinical Investigation 95: 2061–2066. 7738173 10.1172/JCI117892 PMC295794

[10] Schmitz JE, Kuroda MJ, Santra S, Sasseville VG, Simon MA, et al (1999) Control of viremia in simian immunodeficiency virus infection by CD8+ lymphocytes. Science 283: 857–860. 9933172 10.1126/science.283.5403.857

[11] Jin X, Bauer DE, Tuttleton SE, Lewin S, Gettie A, et al (1999) Dramatic rise in plasma viremia after CD8(+) T cell depletion in simian immunodeficiency virus-infected macaques. J Exp Med 189: 991–998. 10075982 10.1084/jem.189.6.991 PMC2193038

[12] Paley MA, Kroy DC, Odorizzi PM, Johnnidis JB, Dolfi DV, et al (2012) Progenitor and terminal subsets of CD8+ T cells cooperate to contain chronic viral infection. Science 338: 1220–1225. 10.1126/science.1229620 23197535 PMC3653769

[13] Utzschneider DT, Legat A, Fuertes Marraco SA, Carrie L, Luescher I, et al (2013) T cells maintain an exhausted phenotype after antigen withdrawal and population reexpansion. Nat Immunol 14: 603–610. 10.1038/ni.2606 23644506

[14] Hong JJ, Amancha PK, Rogers K, Ansari AA, Villinger F (2013) Re-Evaluation of PD-1 Expression by T Cells as a Marker for Immune Exhaustion during SIV Infection. PLoS ONE 8: e60186 10.1371/journal.pone.0060186 23555918 PMC3610666

[15] Salek-Ardakani S, Schoenberger SP (2013) T cell exhaustion: a means or an end? Nat Immunol 14: 531–533. 10.1038/ni.2619 23685816

[16] Birx DL, Loomis-Price LD, Aronson N, Brundage J, Davis C, et al (2000) Efficacy testing of recombinant human immunodeficiency virus (HIV) gp160 as a therapeutic vaccine in early-stage HIV-1-infected volunteers. rgp160 Phase II Vaccine Investigators. J Infect Dis 181: 881–889. 10720508 10.1086/315308

[17] Haubrich RH, Riddler SA, DiRienzo AG, Komarow L, Powderly WG, et al (2009) Metabolic outcomes in a randomized trial of nucleoside, nonnucleoside and protease inhibitor-sparing regimens for initial HIV treatment. AIDS 23: 1109–1118. 10.1097/QAD.0b013e32832b4377 19417580 PMC2739977

[18] Riddler SA, Haubrich R, DiRienzo AG, Peeples L, Powderly WG, et al (2008) Class-sparing regimens for initial treatment of HIV-1 infection. N Engl J Med 358: 2095–2106. 10.1056/NEJMoa074609 18480202 PMC3885902

[19] Doria-Rose NA, Klein RM, Daniels MG, O'Dell S, Nason M, et al (2010) Breadth of human immunodeficiency virus-specific neutralizing activity in sera: clustering analysis and association with clinical variables. J Virol 84: 1631–1636. 10.1128/JVI.01482-09 19923174 PMC2812355

[20] Brenchley JM, Hill BJ, Ambrozak DR, Price DA, Guenaga FJ, et al (2004) T-cell subsets that harbor human immunodeficiency virus (HIV) in vivo: implications for HIV pathogenesis. J Virol 78: 1160–1168. 14722271 10.1128/JVI.78.3.1160-1168.2004 PMC321406

[21] Douek DC, Brenchley JM, Betts MR, Ambrozak DR, Hill BJ, et al (2002) HIV preferentially infects HIV-specific CD4+ T cells. Nature 417: 95–98. 11986671 10.1038/417095a

[22] Kaufmann DE, Kavanagh DG, Pereyra F, Zaunders JJ, Mackey EW, et al (2007) Upregulation of CTLA-4 by HIV-specific CD4+ T cells correlates with disease progression and defines a reversible immune dysfunction. Nat Immunol 8: 1246–1254. 17906628 10.1038/ni1515

[23] Zaunders JJ, Ip S, Munier ML, Kaufmann DE, Suzuki K, et al (2006) Infection of CD127+ (interleukin-7 receptor+) CD4+ cells and overexpression of CTLA-4 are linked to loss of antigen-specific CD4 T cells during primary human immunodeficiency virus type 1 infection. J Virol 80: 10162–10172. 17005693 10.1128/JVI.00249-06 PMC1617311

[24] Colle JH, Moreau JL, Fontanet A, Lambotte O, Joussemet M, et al (2006) Regulatory dysfunction of the interleukin-7 receptor in CD4 and CD8 lymphocytes from HIV-infected patients—effects of antiretroviral therapy. J Acquir Immune Defic Syndr 42: 277–285. 16810123 10.1097/01.qai.0000214823.11034.4e

[25] Watanabe N, Gavrieli M, Sedy JR, Yang J, Fallarino F, et al (2003) BTLA is a lymphocyte inhibitory receptor with similarities to CTLA-4 and PD-1. Nat Immunol 4: 670–679. 12796776 10.1038/ni944

[26] Kinter AL, Godbout EJ, McNally JP, Sereti I, Roby GA, et al (2008) The Common γ-Chain Cytokines IL-2, IL-7, IL-15, and IL-21 Induce the Expression of Programmed Death-1 and Its Ligands. The Journal of Immunology 181: 6738–6746. 18981091 10.4049/jimmunol.181.10.6738

[27] Hathcock KS, Kaech SM, Ahmed R, Hodes RJ (2003) Induction of telomerase activity and maintenance of telomere length in virus-specific effector and memory CD8+ T cells. J Immunol 170: 147–152. 12496394 10.4049/jimmunol.170.1.147

[28] Weng NP, Hathcock KS, Hodes RJ (1998) Regulation of telomere length and telomerase in T and B cells: a mechanism for maintaining replicative potential. Immunity 9: 151–157. 9729035 10.1016/s1074-7613(00)80597-x

[29] Wherry EJ (2011) T cell exhaustion. Nat Immunol 12: 492–499. 21739672 10.1038/ni.2035

[30] Klatt NR, Brenchley JM (2010) Th17 cell dynamics in HIV infection. Curr Opin HIV AIDS 5: 135–140. 10.1097/COH.0b013e3283364846 20543590 PMC2886291

[31] Boswell KL, Paris R, Boritz E, Ambrozak D, Yamamoto T, et al (2014) Loss of Circulating CD4 T Cells with B Cell Helper Function during Chronic HIV Infection. PLoS Pathog 10: e1003853 10.1371/journal.ppat.1003853 24497824 PMC3911819

[32] Quigley M, Pereyra F, Nilsson B, Porichis F, Fonseca C, et al (2010) Transcriptional analysis of HIV-specific CD8+ T cells shows that PD-1 inhibits T cell function by upregulating BATF. Nat Med 16: 1147–1151. 10.1038/nm.2232 20890291 PMC3326577

[33] Topalian SL, Hodi FS, Brahmer JR, Gettinger SN, Smith DC, et al (2012) Safety, Activity, and Immune Correlates of Anti—PD-1 Antibody in Cancer. New England Journal of Medicine 366: 2443–2454. 10.1056/NEJMoa1200690 22658127 PMC3544539

[34] Brenchley JM, Paiardini M, Knox KS, Asher AI, Cervasi B, et al (2008) Differential Th17 CD4 T-cell depletion in pathogenic and nonpathogenic lentiviral infections. Blood 112: 2826–2835. 10.1182/blood-2008-05-159301 18664624 PMC2556618

[35] Gosselin A, Monteiro P, Chomont N, Diaz-Griffero F, Said EA, et al (2010) Peripheral blood CCR4+CCR6+ and CXCR3+CCR6+CD4+ T cells are highly permissive to HIV-1 infection. J Immunol 184: 1604–1616. 10.4049/jimmunol.0903058 20042588 PMC4321756

[36] Perreau M, Savoye AL, De Crignis E, Corpataux JM, Cubas R, et al (2012) Follicular helper T cells serve as the major CD4 T cell compartment for HIV-1 infection, replication, and production. J Exp Med. 10.1084/jem.20121932 PMC3549706 23254284

