# Supplementary figures and images for: Selective Loss of Early Differentiated, Highly Functional PD1high CD4 T Cells with HIV Progression
Source: PLoS One. 2015 Dec 17;10(12):e0144767. doi: 10.1371/journal.pone.0144767 (PMC4692060; doi:10.1371/journal.pone.0144767)

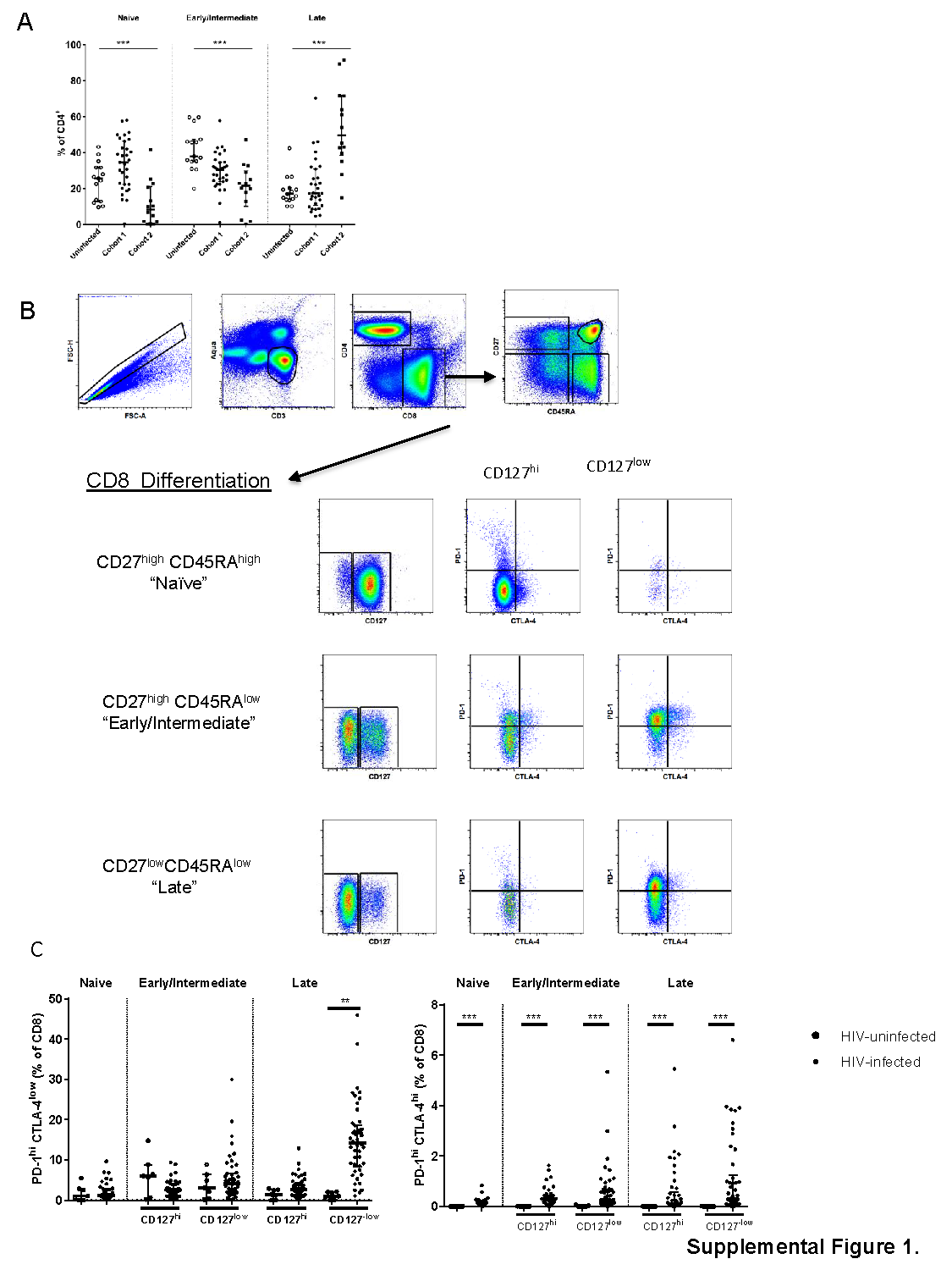

Supplement: S1 Fig — (A) Distribution plots showing skewed CD4 differentiation of HIV- infected subjects compared to HIV-uninfected (open circles, n = 15) from two cohorts with HIV infection: Cohort 1 (median CD4 count 525 cells/μl, filled circles, n = 31); and Cohort 2 with more advanced infection (median CD4 count 148 cells/μl, filled squares, n = 14). (B) Representative flow cytometry plots and the gating strategy used to characterize CD4 and CD8 T cell populations. (C) Comparative plots of PD-1 and PD-1/CTLA-4 expression by differentiation status and CD127 staining for populations of Naive (CD27high CD45RAhigh), Early/Intermediate (CD27highCD45RAlow) and Late (CD27low CD45RAlow) CD8 T cells from HIV-uninfected (open circles, n = 9) and HIV-infected (filled circles, n = 31) subjects. *p< 0.05, **p< 0.001, ***p< 0.0001 by Mann-Whitney test. (TIFF) [file pone.0144767.s001.tiff]

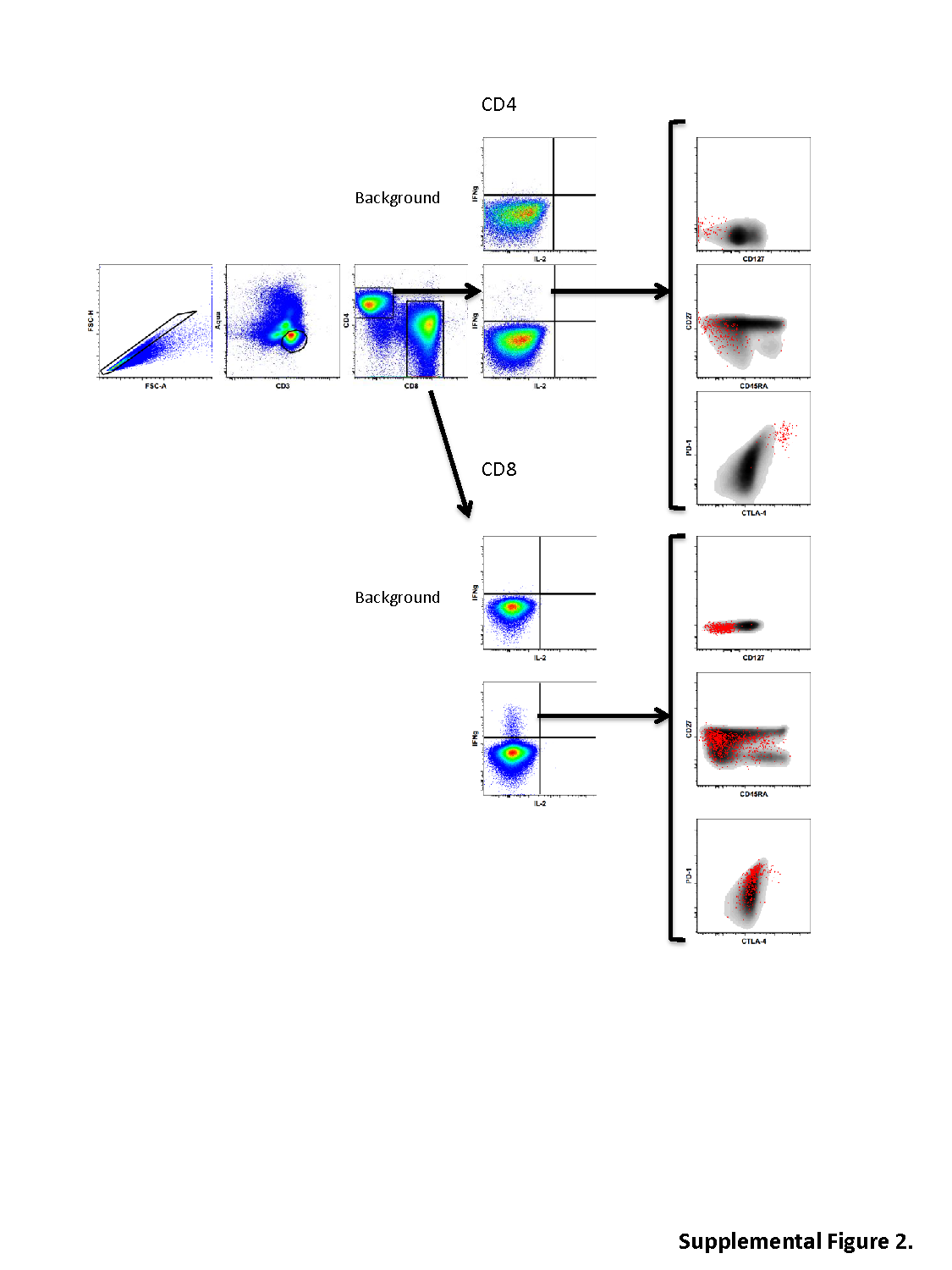

Supplement: S2 Fig — Representative flow cytometry, gating strategy and overlay plots of Gag-specific, IFN-g-producing CD4 and CD8 T-cells for specific populations is shown. (TIFF) [file pone.0144767.s002.tiff]

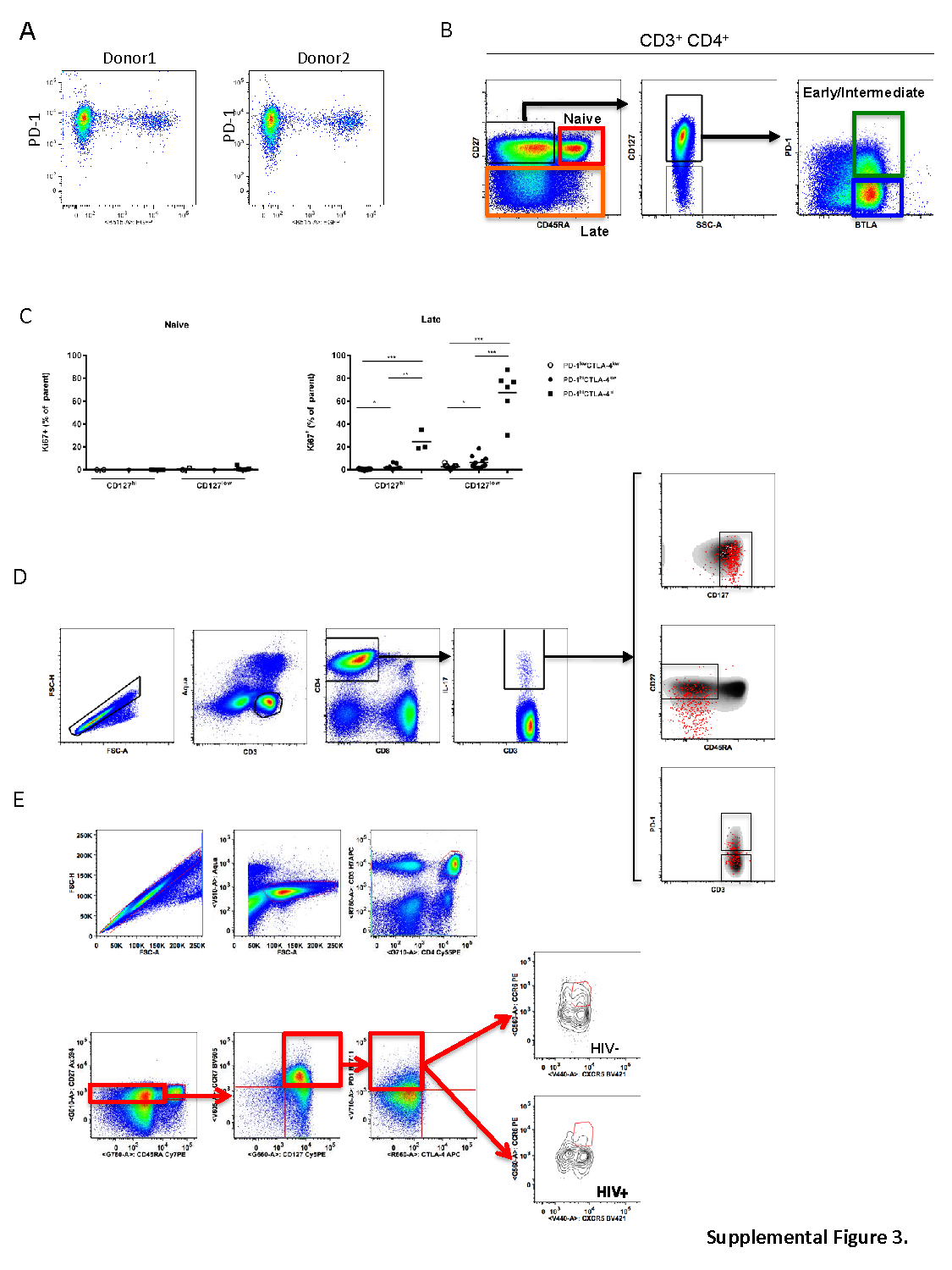

Supplement: S3 Fig — (A) Sorted memory (Early/Intermediate, CD27high CD45RAhigh) CD4 T cells from two healthy donors were subjected in vitro HIV infection. PD-1 levels in non-infected (EGFP-) and cells harboring virus (EGFP+) were analyzed by flow cytometry. (B) Gating strategy for sorting PD-1highCD127high Early/Intermediate and other CD4 T cell populations. Due to the requirement for surface staining, intracellular anti-CTLA-4 was not included as sorting parameter. (C) Percent Ki67+ staining cells for CD127high and CD127low naïve and late CD4 T cells from HIV-infected Cohort 1 (n = 11). Not all populations for all donors are plotted due to the small population size.. (D) Representative flow cytometry, gating strategy and overlay plots after polyclonal stimulation with SEB for IFN-g or IL-17 (shown) producing CD4 T-cells for specific populations is shown. (E) Representative flow cytometry plot and gating strategy demonstrating loss of CD127highCCR7highPD-1highCTLA-4low CXCR5highCCR6high Early/Intermediate CD4 T cells with HIV infection. (TIFF) [file pone.0144767.s003.tiff]
